# Skeletal Plasmacytoma: Progression of disease and impact of local treatment; an analysis of SEER database

**DOI:** 10.1186/1756-8722-2-41

**Published:** 2009-09-24

**Authors:** Muhammad Umar Jawad, Sean P Scully

**Affiliations:** 1 Departments of Orthopaedics, University of Miami Miller School of Medicine, 1400 NW, 12th Avenue, Miami, FL 33136, USA

## Abstract

**Background:**

Previous reports suggest an as yet unidentifiable subset of patients with plasmacytoma will progress to myeloma. The current study sought to establish the risk of developing myeloma and determine the prognostic factors affecting the progression of disease.

**Methods:**

Patients with plasmacytoma diagnosed between 1973 and 2005 were identified in the SEER database(1164 patients). Patient demographics and clinical characteristics, treatment(s), cause of death, and survival were extracted. Kaplan-Meier, log-rank, and Cox regression were used to analyze prognostic factors.

**Results:**

The five year survival among patients initially diagnosed with plasmacytoma that later progressed to multiple myeloma and those initially diagnosed with multiple myeloma were almost identical (25% and 23%; respectively). Five year survival for patients with plasmacytoma that did not progress to multiple myeloma was significantly better (72%). Age > 60 years was the only factor that correlated with progression of disease (p = 0.027).

**Discussion:**

Plasmacytoma consists of two cohorts of patients with different overall survival; those patients that do not progress to systemic disease and those that develop myeloma. Age > 60 years is associated with disease progression. Identifying patients with systemic disease early in the treatment will permit aggressive and novel treatment strategies to be implemented.

## Introduction

Plasmacytoma results from clonal proliferation of plasma cells that are identical to plasma cells of myeloma on both the cyotologic and immunophenotypic levels. Plasmacytoma can be subclassified as osseous disease or extraosseous tumor [1-7]. The clinical presentation of these diseases represent different groups of patients in terms of location, tumor progression, and overall survival rate [8]; however, they share many of the biologic features of other plasma cell disorders [1,9]. Skeletal plasmacytoma is characterized clinically by a radiolytic lesion involving any part of the skeleton, a clonal plasma cell infiltrate and an absence of disseminated bone marrow involvement.

Local radiotherapy and alternatively surgery are treatment options yielding adequate local control [7,10]. Despite local treatment efforts, 50-60% of patients with plasmacytoma progresses to myeloma [2,11,12]. It has been reported that skeletal plasmacytoma is known to progress more frequently to multiple myeloma than extraskeletal disease [2,11,13]. Most of the basis for the natural history of plasmacytoma is derived from reports emanating from single institutions. Knobel et al and Kilciksiz et al reported multi-center experiences but the small number of patients limited the statistical power of these studies[10,14]. Recently Dores et al reported incidence and survival for patients with plasmacytoma, extraskeletal disease and myeloma from the SEER database [15]. In this study there was no analysis of prognostic factors associated with neither progression of cases initially diagnosed as plasmacytoma into myeloma nor an assessment of local disease control.

The current manuscript attempts to ascertain the impact of local treatment on disease progression in patients with skeletal plasmacytoma. Specifically, the study sought to establish the incidence of development of myeloma and the treatment outcomes of patients initially diagnosed with plasmacytoma. Furthermore, the study seeks to compare clinical parameters in cases of plasmacytoma not progressing to systemic disease with those developing into myeloma to identify prognostic factors. In order to answer these questions, the SEER Database was utilized as a source of patient data. Previously the population based SEER database has been used to describe the outcomes for breast, colorectal, prostate, lung, ovarian, sarcomas and neuroectodermal cancers and has been validated as to the accuracy [16-21]. The current study investigates the impact of local treatment on skeletal plasmacytoma and its progression to myeloma and demonstrates that a younger patient population has localized disease which does not progress to systemic disease.

## Methods

The Surveillance, Epidemiology, and End-Results (SEER) Program of the National Cancer Institute (NCI) was established as a direct result of the National Cancer Act 1971. Currently SEER collects data from 17 population based registries covering approximately 26% of the US population. It is the only comprehensive source of population based data in the U.S. that includes the stage of cancer at the time of diagnosis and follow-up of all patients for survival data. In addition, each registry collects data on patient demographics, primary tumor site and morphology, and first course of treatment (occurring within 4 months of diagnosis) [22-34]. The SEER program is currently regarded as the standard of quality among cancer registries around the world with case completeness of 98% [35].

The SEER database was used to identify all cases of skeletal plasmacytoma diagnosed from 1973-2005 using International Classification of Disease for Oncology, 3^rd ^Edition (ICD-O-3) [36]. We used primary site of lesion to specifically select for cases involving bone. A total of 1164 patients were identified and information regarding patient demographics, clinical characteristics, treatment related characteristics were extracted (if provided within 4 months of diagnosis), and survival time (months) until death or loss to follow-up. Percentages were based on available data for each individual variable. Patients with missing data were excluded from each respective univariate and multivariate analysis.

Patients' age was converted to a categorical variable (0-29, 30-59, >60) for the purpose of analysis. The appendicular skeleton included long and short bones of limbs and associated joints, and the scapula. Axial skeleton included vertebra, ribs, sternum, clavicle and associated joints, bones of skull, face and associated joints, mandible, and pelvic bones. Staging categories of local, regional, and distant were described in SEER according to AJCC staging system [37]. Since, skeletal plasmacytoma by definition is a localized disease process we excluded the cases (5.6% of total) designated as 'distant'. Year of diagnosis was categorized in four categorical variables: 1973-1975, 1976-1985, 1986-1995, and 1996-2005. Results reported herein are in compliance with the Health Insurance Portability and Accountability Act of 1996.

Incidence rates were age adjusted and normalized using the 2000 US Standard population [38]. Statistical analysis was performed using SPSS version 17.0 (SPSPSS Inc., Chicago, IL). Chi-square test was used to make correlations between categorical variables. Overall and disease-specific survival from the time of initial diagnosis to the date of last contact (or the date of death) was calculated using the Kaplan-Meier method. The effects of demographic, clinical, pathological, and treatment variables were tested using the log-rank test for categorical values. A multivariate analysis was carried out for determination of independent prognostic factors using the Cox proportional hazards model. All prognostic factors found to be significant in the univariate analysis, namely gender, stage, primary site, size, and surgical therapy were included in a multivariate analysis.

## Results

A total of 1164 cases of plasmacytoma are included in the SEER Data-base from 1973-2005. Nearly three quarter of the cases (74.7%) were diagnosed during the most recent decade, 1996-2005 reflecting the geographic expansion of the data collection effort during these years. The demographic and clinical characteristics of the entire patient cohort are summarized in Table 1. Most patients were older than 60 years at diagnosis (63.7% of the cases). Males comprised 61.9% of patients; race was predominantly Caucasian (84.1%) and ethnicity predominantly non-Hispanic (89.7%), respectively. Among all the cases identified, 5.2% of the tumors were designated as 'distant' at the time of diagnosis and thus were excluded from further analysis. Staging information was available for 51.4% of the cases that were subjected to analysis. Surgical resection alone was performed in 34.6% of patients, solely radiation therapy administered in 53.9%, and combined surgery and radiation in 0.9%. Approximately 10.5% of patients received no therapy for local disease control (dns).

**Table 1:** Demographic and clinical characteristics of the entire patient cohort

		***n***	**Valid % of total**
**Total Patients**		1,164	100
**Age**			
	0-29	13	1.1
	30-59	410	35.2
	>60	741	63.7
**Gender**			
	Male	720	61.9
	Female	444	38.1
**Race**			
	White	973	84.1
	Black	135	11.7
	Other	49	4.2
**Ethnicity**			
	Hispanic	119	10.3
	Non-Hispanic	1,035	89.7
**Stage**			
	Local	598	100
**Lesion**			
	Single	960	82.5
	Two	180	15.5
	>Two	24	2.1
**Location**			
	Bone NOS	15	1.3
	Appendicular	187	16.1
	Axial	962	82.6
**Surgery**			
	Yes	404	34.9
	No	752	65.1
**Radiation**			
	Yes	927	80.9
	No	219	19.1
**Year of Diagnosis**			
	1973-1975	2	0.2
	1976-1985	27	2.3
	1986-1995	265	22.8
	1996-2005	870	74.7

The overall incidence for plasmacytoma was 0.3462/100,000 in 2005; similar to what has been reported by Dores et al [15]. Afro-American to Caucasian Incidence Rates Ratio to develop plasmacytoma was found to be approximately 1.30 (dns). Five and ten year overall survival for patients with skeletal plasmacytoma is summarized in Table 2. Kaplan-Meier prediction of survival of patients with plasmacytoma is affected by race with "races other than Caucasians" and Afro-Americans faring significantly better than Caucasians (p < 0.001). Since other races made up only 4.2% of the entire patient cohort, thus this result should be interpreted with caution. There was no significant difference in outcome between Caucasians and Afro-Americans.

**Table 2:** Disease-specific survival according to demographic and clinical characteristics (proportion surviving)

		**5-Year Survival**	**10-Year Survival**	**p-value**
**Overall**		0.57	0.37	n/a
**Age**				
	0-29	0.9	0.9	
	30-59	0.8	0.63	
	>60	0.45	0.23	<0.001*
**Gender**				
	Male	0.59	0.4	0.008
	Female	0.54	0.32	
**Race**				
	White	0.56	0.36	
	Black	0.56	0.37	
	Other	0.76	0.64	0.001**
**Ethnicity**				
	Hispanic	0.65	0.47	0.152
	Non-Hispanic	0.56	0.36	
**Stage**				
	Local	0.54	0.38	n/a
**Lesion**				
	Single	0.59	0.39	0.032***
	Two	0.53	0.36	
	> Two	0.36	0.15	
**Location**				
	Bone NOS	n/a	n/a	
	Appendicular	0.52	0.33	
	Axial	0.58	0.39	0.240****
**Surgery**				
	Yes	0.59	0.4	0.286
	No	0.56	0.36	
**Radiation**				
	Yes	0.6	0.39	<0.001
	No	0.46	0.31	
**Year of Diagnosis**				
	1973-1975	1	1	
	1976-1985	0.63	0.44	
	1986-1995	0.52	0.32	0.072*****
	1996-2005	0.59	0.42	

*P *value shown for Log rank test between variables; *p < 0.001 for age 0-29 vs. >60 and. 30-59 vs. >60, 30-59 vs. 0-29: p = 0.158; **p = 0.001 for White vs. Other only, White vs. Black: p = 0.932, and Black vs. Other: p = 0.006; ***p = 0.032 for Single vs. >Two only, Single vs. Two: p = 0.275, Two vs. > Two only: p = 0.182; ****p = 0.240 for Appendicular vs. Axial only, Bone NOS vs. Appendicular: p < 0.001, Bone NOS vs. Axial: p < 0.001; *****p < 0.072 for 1973-1975 vs. 1986-1995 only, 1973-1975 vs. 1976-1985: p = 0.125, 1973-1975 vs. 1996-2005: p = 0.109, 1976-1985 vs. 1986-1995: p = 0.486, 1976-1985 vs. 1996-2005: p = 0.365, 1986-1995 vs. 1996-2005: p = 0.194.

**Figure 1:**
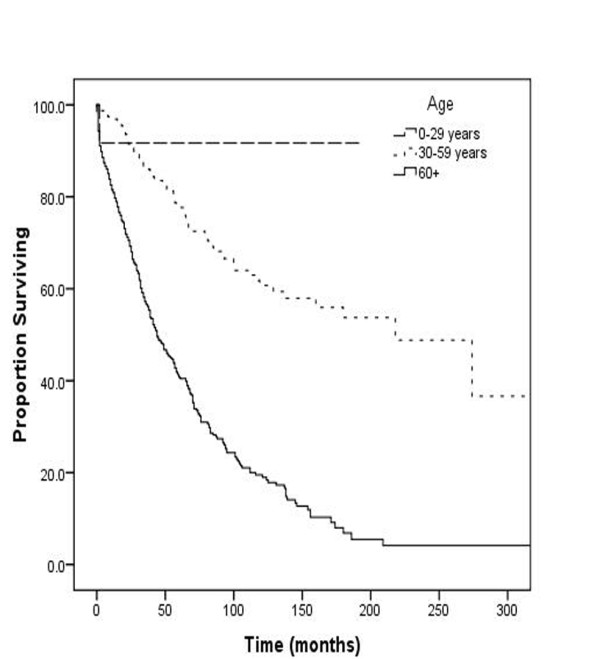
**Overall Survival stratified by Age**.

Patients diagnosed at age < 60 years (Figure 1) had a significantly better 5 year survival (90% for patients aged 0-29 years & 80% for patients 30-59 years) when compared to patients diagnosed at age >60 years (5 year survival 45%) (p < 0.001). Patients with skeletal plasmacytoma had an overall survival of 57% at 5 years and 37% at 10 years. Females have a significantly lower 5 and 10 year survival than males (5 year survival 54% v/s 59% for males (p-value = 0.008). Patients with a solitary lesion carried significantly better prognosis with a 5 year survival of 59% as compared to 36% for patients with more than 2 lesions (p = 0.032). There was no significant difference in survival between patients with a single or two lesions (p-value = 0.28). There was no significant difference in 5 year survival outcome for axial lesions (58%) as compared to appendicular lesions (52%) (p = 0.24).

**Figure 2:**
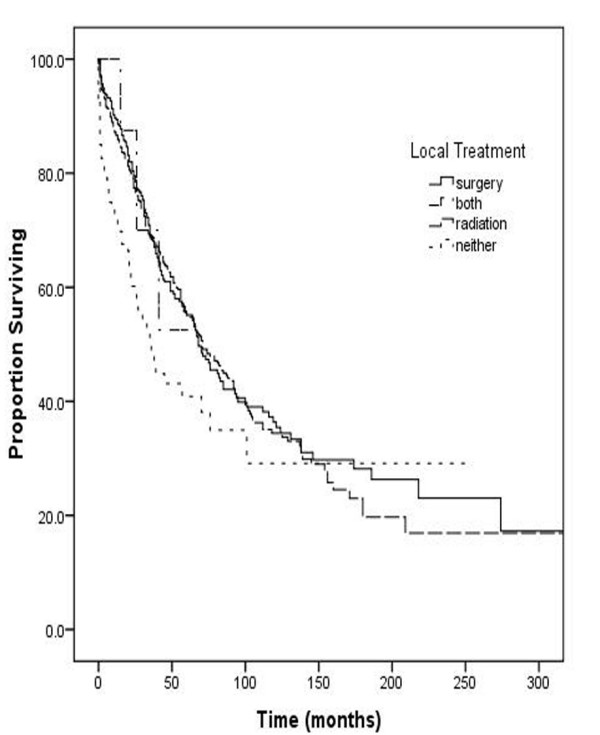
**Overall Survival stratified by Local Treatment**.

Patients undergoing surgery fared no better than patients without resection (5 year survival 59% and 56% respectively, p = 0.29). In contrast, administration of radiation therapy was associated with an improvement in survival (5 year survival of 60% among patients with radiation therapy as compared to 46% without any radiation therapy, p < 0.001). Further analysis revealed that use of any local disease control modality, either surgery or radiation therapy was associated with better outcomes as compared to the absence of any local disease control (p < 0.001; Figure 2). Surgery and radiation were found to be equally effective in local disease control. Use of both modalities was not associated with any significant survival advantage. No significant improvement in survival could be observed over time when stratified by decade for the past two decades (p = 0.19).

Table 3 illustrates a step-wise multivariate analysis employing the Cox proportional hazard model to ascertain the independent significant variables for the entire cohort. The parameters age > 60 years (p < 0.001), race other than Caucasians and Afro-Americans (p < 0.05), and absence of radiation therapy (p < 0.001) were all independent predictors of lower overall survival.

**Table 3:** Cox proportional hazards model for risk of death from Ewing's Sarcoma

		***n***	**Hazard ratio**	**95% CI**	**p-value**
**Age**					
	0-29	13	0.075	0.011-0.537	0.01
	30-59	401	0.277	0.220-0.349	<0.001
	>60	726		Reference Group	
**Gender**					
	Male	710	0.918	0.764-1.103	0.362
	Female	430		Reference Group	
**Race**					
	White	957	1.893	1.037-3.455	0.038
	Black	134	2.080	1.086-3.985	0.027
	Other	49		Reference Group	
**Lesions**					
	Single	939	0.851	0.523-1.387	0.518
	Two	177	0.85	0.504-1.434	0.542
	> Two	24		Reference Group	
**Radiation**					
	Yes	925	0.647	0.521-0.804	<0.001
	No	215		Reference Group	

CI: Confidence Interval

**Figure 3:**
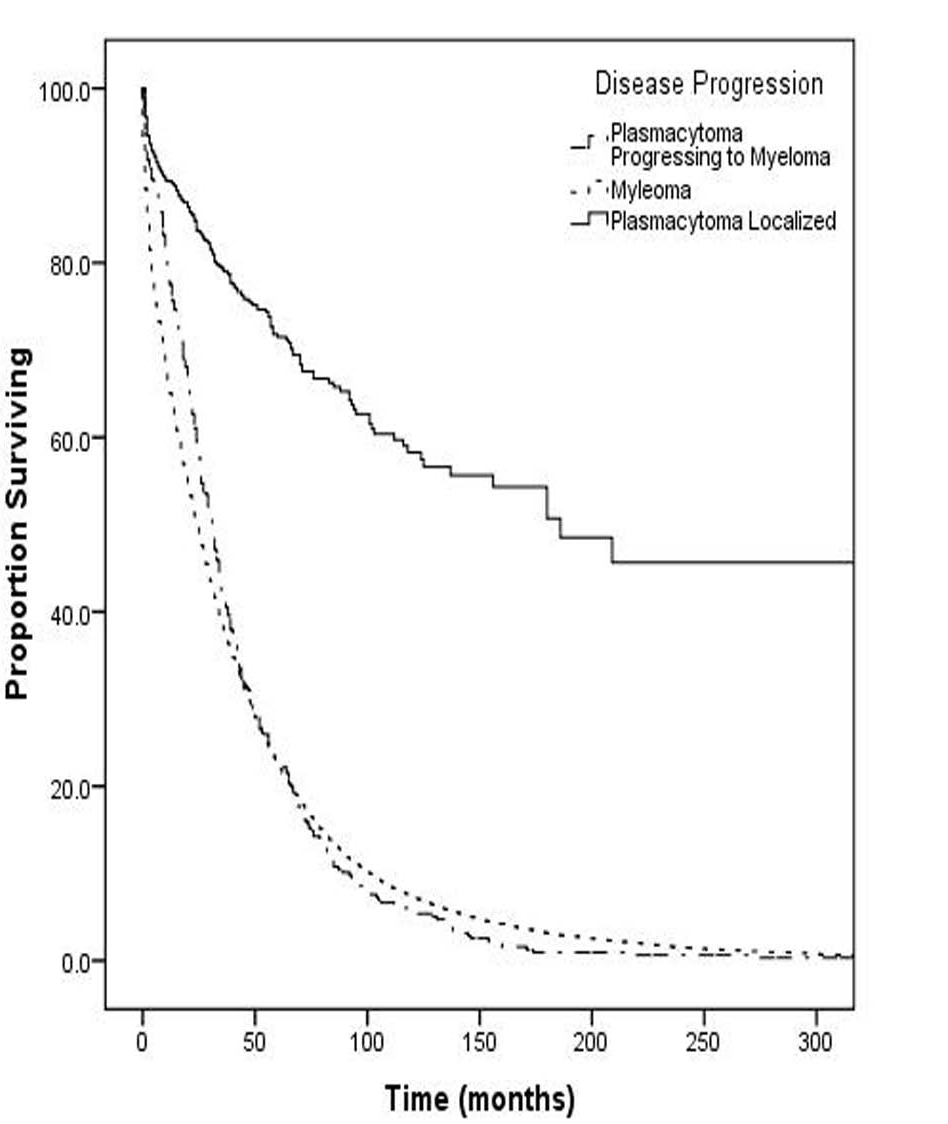
**Overall Survival stratified by Disease Progression**.

In order to compare the plasmacytoma as a localized disease with cases progressing to myeloma, the survival and cause of death information were extracted regarding all the cases of multiple myeloma from 1973-2005 (54,244 cases). We found that there was no significant difference in 5 year survival among patients initially diagnosed with multiple myeloma and those initially labeled as plasmacytoma who subsequently later progressed to multiple myeloma (5 year survival of 25% and 23% respectively). In contrast, 5 year survival for patients with plasmacytoma who did not progress to multiple myeloma was significantly better with (p < 0.001 5 year survival = 72%), Figure 3. Some of the more frequent causes of death in patients with plasmacytoma not progressing to myeloma included Diseases of Heart (4.5%) and Cerebrovascular Diseases (1.0%) as would be expected for patients in this age group.

Analysis of statistically significant factors on multivariate analysis did not reveal any prognostic factors significantly associated with progression of plasmacytoma into a systemic disease other than age (Table 4). Association between Age >60 years and development of myeloma approached significance (p = 0.027).

**Table 4:** Prognostic Factors Associated with Progression

		**Myeloma**	**Other Causes**	**Chi-Square**	
**Age**					
	0-29 years	0	1	0.027	
	30-59 years	71	31		
	60+ years	245	188		
**Race**					
	White	277	191	0.457	
	Black	34	22		
	Others	5	7		

In order to assess the potential ascertainment bias: the potential bias of falsely diagnosing patients with myeloma as plasmacytoma patients, a cross-tabulation was performed between cause of death and different treatment groups. Our analysis reveals 27 of 60 patients with no attempt at local control, died of myeloma. This ratio of progression to myeloma was similar in other treatment groups (dns) with chi-square not revealing any statistical significance.

## Discussion

The current study is the first to demonstrate the impact of local treatment on skeletal plasmacytoma using a population based registry. It further demonstrates the differences in treatment related outcomes among patients diagnosed with plasmacytoma not progressing to myeloma, those that progressed to myeloma. Epidemiological studies comparing SEER areas to non-SEER areas in the U.S. conclude that their age and sex distributions are comparable except that SEER areas tended to be more affluent and more urban than non-SEER areas [34]. When compared to NPCR and USCS, incidence rates for all sites combined were lower in SEER. But for category of interest: 'Bones and Joints', the differences were small: 0.6 in SEER versus 0.8 per 100,000 in NPCR among Black males was the largest reported difference [34]. Despite adequate local control with radiotherapy and/or surgery, cause of death in 59% of the patients initially diagnosed with plasmacytoma was progression to myeloma. Similarly, Soutar et al. reported >75% progression to multiple myeloma for skeletal plasmacytoma [12]. In the current study it cannot be determined that what is the absolute rate of progression to multiple myeloma among patients diagnosed with plasmacytoma, since some of the patients died of other causes and hence are censored. Despite these limitations, we can confidently say that rate of progression to Multiple Myeloma among cases of plasmacytoma is at least 59% and may be slightly greater than this number.

Also, the 5 year survival for patients diagnosed with multiple myeloma and those diagnosed as plasmacytoma initially but who subsequently developed myeloma later on, were almost identical (Figure 3). This may suggest an underlying misdiagnosis for cases of myeloma as plasmacytoma. In order to overcome this problem, the use of MRI in initial staging of plasma cell neoplasm has been advocated by some authors [39] while others have reported conflicting evidence [14]. We tried to address this issue by determining any significant association of the independent predictors of overall survival among patients with plasmacytoma and subsequent development of multiple myeloma. None of the factors considered revealed any significant association with development of myeloma, other than age. Age > 60 years was significant with a p = 0.027 (Table 4). Conflicting evidence for age as a predictor of progression to myeloma has been reported in literature, with some studies supporting this observation [3,40-42] while others have found no association [43-45]. Kilciksiz et al. identified age as an independent prognostic factor for progression to myeloma [10].

Another controversy highlighted in literature involves the fate of patients with plasmacytoma in regards to disease progression. While some have demonstrated a significant portion of patients free of disease after 5 and 10 years, others believe in the inevitability of progression for all patients [41,43,46,47]. The current study clearly demonstrates that there is a cohort of plasmacytoma patients that do not progress to myeloma demonstrate as high as 72% 5-year survival rate (Figure 3).

Surgery alone has been proposed as the best treatment option for extramedullary plasmacytomas [48], but conflicting evidence has also been provided in the literature [14]. Uni- and multivariate analysis in the current study revealed no survival advantage associated with surgery alone for skeletal plasmacytoma (Table 2). This result reflects a selection bias: relatively smaller number of patients underwent surgical resection as a local treatment option. Radiotherapy was employed for a majority of cases. Further analysis, after stratification based on local treatment option clearly demonstrates that use of either option, i.e. surgical therapy or radiotherapy is associated with an improvement in survival in skeletal disease. Neither of the treatment options showed a survival benefit over the other nor similarly employment of both modalities of local control did not further improve survival.

Limitations of the current study include lack of any information on specific chemotherapy regimens or any other adjuvant therapy in the SEER Database. Thus we are unable to comment directly on survival benefit conferred upon by the use of chemotherapy or efficacy of a particular regimen in a particular subset of patients. Similarly, no information regarding any medical history, radiological studies or serological work up is provided in the database limiting our analysis on diagnostic accuracy of MRI, and prognostic significance of absence of myeloma protein, anemia, hypercalcemia, renal insufficiency and stability of M-protein. Accuracy of staging information can be a potential pitfall in all studies based on database. Another issue associated with the reported data in SEER is the potential bias of falsely labeling myeloma patients as plasmacytoma patients. The results of bone marrow aspirate are not reported in SEER, and in clinical practice treatment decisions are usually made based on this parameter. This raises the suspicion of marrow involvement >30% among those not receiving the local therapy. Our analysis reveals no correlation between any treatment groups and 'cause of death'. We also attempted to address the controversy regarding progression to myeloma. In clinical practice, the evaluation regarding progression to myeloma is carried out after a specified time interval. SEER does not report the time for evaluation regarding progression; we assessed the outcome using 'cause of death' information. The time to progression cannot be assessed.

## Conclusion

Despite the above mentioned limitations such as retrospective nature of data, lack of information about chemotherapy or serological or radiological investigations; the current study addresses some of the controversies in disease progression and impact of local treatment for plasmacytoma using a large populations based well validated national database. The current study also reiterates the need of better understanding of the disease process of plasma cell neoplasm and the inclusion of molecular markers in the database.

## Competing interests

The authors declare that they have no competing interests.

## Authors' contributions

MUJ carried out analysis and wrote the manuscript.

SPS conceived the idea and was the senior author in preparation of manuscript.

Both authors have read and approve the manuscript.
